# Comparisons between context-specific and beverage-specific quantity frequency instruments to assess alcohol consumption indices: Individual and sample level analysis

**DOI:** 10.1371/journal.pone.0202756

**Published:** 2018-08-17

**Authors:** Polathep Vichitkunakorn, Karnsunaphat Balthip, Alan Geater, Sawitri Assanangkornchai

**Affiliations:** 1 Department of Family Medicine and Preventive Medicine, Faculty of Medicine, Prince of Songkla University, Hat Yai, Songkhla, Thailand; 2 Epidemiology Unit, Faculty of Medicine, Prince of Songkla University, Hat Yai, Songkhla, Thailand; 3 Faculty of Nursing, Prince of Songkla University, Hat Yai, Songkhla, Thailand; La Trobe University, AUSTRALIA

## Abstract

There are many survey instruments to determine drinking patterns and alcohol consumption levels in the general population. This study aims to compare the context-specific quantity-frequency (CSQF) and beverage-specific quantity-frequency (BSQF) methods to estimate alcohol consumption indices at individual and sample levels. A community-based cross-sectional study was conducted among a population aged 15 years and older in Songkhla Province, Thailand. The BSQF and CSQF questionnaires with a 3-month retrospective time frame and in random order were applied to each participant. The CSQF was developed to ask more about the drinking contexts. The questions elicited information on location, partner, beverage, quantity, and frequency for five common drinking situations. Among 804 participants, 183 drank alcohol in the last three months. At the individual level, total alcohol consumption of almost all types of beverage by the CSQF was higher than the BSQF in approximately 50% of current drinkers and was mainly accounted for by the higher report of average quantity. At the sample level, there were no significant differences in the average daily intake, 3-month intake per drinker or per capita consumption between instruments. The interview duration and burden of answering the questions by the participants for the CSQF were not significantly higher than those for the BSQF. In summary, the fuller picture of drinking behaviors from the CSQF has several valuable methodological advantages and provides information allowing alcohol policies to be more directly specific to certain target populations or situations. The CSQF is a prototype questionnaire and forms the basis for a contextual approach. However, additional methodological studies need to be explored.

## Introduction

Drinking alcohol is a causal factor in many injuries and disease conditions [1, 2]. The harmful use of alcohol ranked among the top five modifiable risk factors for morbidity and mortality throughout the world [3]. Alcohol consumption results in substantial societal costs through loss of productivity, healthcare expense, criminal activity, and violence [4].

In Thailand, the average recorded annual alcohol per capita consumption (APC) in 2014 was 7.1 liters of pure alcohol per person aged 15 years or older, ranking 4^th^ in Asia following the Republic of Korea (12.3), Laos (7.3), and Japan (7.2). The National Health and Welfare survey 2015 found that most Thai drinkers (60% of all drinkers) are occasional drinkers. Drinking is seasonal and also varies by days of the week, holidays, and other special events (e.g., cultural events, birthday, or Buddhist Lent). Only 3.4% of Thai drinkers (6.1% of male and 1.0% of female) were medium- to high-risk drinkers (>40 g/day for males, >20 g/day for females).

An accurately measured alcohol consumption survey provides information on the levels, patterns, and contexts of alcohol consumption and alcohol-related harm and can help to determine relevant harm reduction interventions [5]. The WHO recommended that alcohol survey components include the volume of alcohol consumed, drinking pattern, and drinking context (e.g., festive drinking, the proportion of drinking events when getting drunk, drinking with meals, drinking in a public place, and drinking intensity) [6].

There are several alcohol consumption measuring instruments, which have strong and weak points for capturing the volume, pattern, and context of drinking at individual and population levels. The beverage-specific quantity-frequency (BSQF) instrument is a modification of the quantity-frequency (QF) instrument, which is the most widely applied and simplest method. The QF method estimates the usual frequency and amount of drinking in a 30-day or one-year time frame, whereas the BSQF asks for their usual frequency and amount of each specific beverage. The BSQF instrument has some advantages over the traditional QF instrument in terms of increasing recall ability [7], higher total volume estimation [8], and average daily intake [9].

Contextual factors are associated with drinking behaviors and consequences such as drinking events (e.g., weekend, holiday, and cultural event), partners (e.g., friends, family, and strangers), and location (e.g., house, pub/bar, restaurant, and workplace) [10–12]. For instance, a higher level of alcohol consumption was associated with going on spring break trips with friends among college students [13]. Drinking at a large party or having parents who provide alcohol were associated with heavy episodic drinking [11]. Also, there are complex relationships between alcohol-related harm and alcohol consumption. A neglect of the drinking contexts in these relationship pathways could lead to a misinterpretation of the association between alcohol consumption and harm in the sense that some drinking patterns are more harmful than others [14].

The last few decades have seen a trend of increased use of a contextual approach technique in alcohol surveys. The specific social context technique for alcohol consumption survey was originally developed in 1973 [15]. After that, there have been many studies that focused on the contextual approach, for example, different drinking locations (home, bar, restaurant) [16, 17], times (work days, weekends) [18], and situations (evening meal in a restaurant, organization meeting, party at home, picnic, while watching television, and while spending a quiet evening at home) [19, 20]. Lastly, a within-location beverage-specific consumption instrument, which was developed by International Alcohol Control Study (IAC) in New Zealand and used to estimate the APC, accounted for 94% of the estimated taxable alcohol available for consumption [21].

Despite of the high benefits of the contextual approach in specifying drinking situation, there are some setbacks of this approach, for example, double counting of the overlapping drinking events [20, 22] and response burden when a respondent has to answer several loops of questions for various drinking situations [22]. Thus, this makes such approach less used in general population surveys, and studies comparing the context-specific technique to the traditional BSQF have not been available.

In this study we developed a context-specific quantity-frequency (CSQF) questionnaire that aimed to accurately measure alcohol consumption using questions that probe the context of drinking. The CSQF will be useful to identify drinking environments associated with high-risk alcohol consumption in a general population survey.

The two methods used most often to test the validity of consumption are convergent validity and a comparison with data on taxable alcohol available (sales data) for consumption [21]. The convergent validity method assesses the individual level consistency between these measures and the other survey measures (i.e. correlation of alcohol consumption between two measurement tools). The comparisons with taxable alcohol available for consumption are derived from data on production, import/export from the revenue department, and taxation office. Most studies found that the results from a population-based survey could account for 40% to 60% of taxable alcohol available [23–25].

This study aimed to compare the CSQF and BSQF to estimate total alcohol consumption at the individual level and average daily intake, 3-month per drinker, and 3-month per capita consumption at the sample level.

## Materials and methods

### Study design and population

A community-based cross-sectional study was conducted among a population aged 15 years and older. We recruited current drinkers and non-current drinkers that included lifetime abstainers and former drinkers who drank but had not drunk during the previous three months because we desired to assess the 3-month per capita consumption at the sample level as well. A multistage sampling technique was used. In the first stage, four sub-districts in both urban and rural areas in Songkhla Province in southern Thailand were selected randomly. In the second stage, eight villages were selected with probability proportional to size. In the third stage, households within each village were listed and 50 to 52 households were selected by systematic random sampling procedure. In the fourth stage, two participants in each household were selected using the Kish selection grid [26]. The final sample comprised 804 participants. Although 818 people were selected, only 804 agreed to participate in the interview which resulted in the response rate of 98.3%.

### Data collection and instruments

A structured questionnaire covering demographic characteristics and alcohol consumption was used. A face-to-face interview with paper-and-pencil administration was performed by trained interviewers. The actual time spent in completing the survey was measured by recording the starting and ending points. Furthermore, the perceived burden of the respondents to answer each instrument was measured using a 5-point rating scale: *“Did you find it easy or burdensome to answer the questions*?*”*

The alcohol consumption part comprised two instruments: the BSQF and the CSQF. A retrospective time frame of three months was set for both instruments. The instruments were employed in a random sequence to diminish recall bias. Pictures of various kinds of alcoholic beverages and containers were used to increase recall ability of the alcohol volume consumed by the respondents.

The BSQF asked three questions separately for each specific beverage consumed in the previous 3 months. The first question determined the frequency level and the other questions defined the usual amount of each beverage actually consumed. The CSQF instrument (item 5 to 7 of the CSQF) used a similar question format and response categories as the BSQF (item 2 to 4 of the BSQF). However, it asked more about the drinking context (item 2 to 4 of the CSQF). The questions elicited information on location, partner, beverage, quantity, and frequency for each common drinking situation or event (Table 1). The CSQF can provide a maximum of three drinking locations in each situation, with a total of five drinking situations. So, each participant had the chance to respond to 15 types (3×5 = 15) of drinking events. A drinking event was a unique combination of one specified drinking situation, location, drinking partner(s), beverage type(s), and volume consumed.

**Table 1:** Questions used for the BSQF and CSQF instruments.

Instrument	Question	Answer
**BSQF**	1. “During the last 3 months, did you drink these kind of beverages*?”	
	2. How often did you usually have … (for specified beverage) … in the last three months?”	• Every day • 5 to 6 days/week • 3 to 4 days/week • 1 to 2 days/week •1 to 3 days/3 month (can choose one frequency category).
	3. On those days when you had … (for specified beverage)…, which containers did you usually use?	The interviewer shows pictures of various kinds of containers to the interviewee (can choose one drinking container type).
	4. And, how much did you usually have … (for specified beverage)… per day in that container?	Answered in terms of the number of containers (can answer only one number).
These four questions were asked in a loop for seven common kinds of beverages* ***(i*.*e*., *beer*, *white spirits*, *whisky*, *local beverage*, *wine*, *wine coolers and vodka)*** ***Pictures of beverage in each category were provided*.**		
**CSQF**	1. “During the last 3 months, did you drink in these situations**?”	
	2. Where did you usually drink … (for specified situation)… in the last three months?	Own house, someone else’s house, restaurant, pub/bar, workplace, religious place, local shop (can choose a maximum of three locations for each situation).
	3. With whom did you usually drink in … (for each unique combination of situation(s) and location(s))…?”	Alone, family, male friends, female friends, strangers, colleagues (can choose one drinking partner(s)).
	4. What beverage did you usually drink at … (for each unique combination of situation(s), location(s), and partner(s)) …?	The interviewer provides pictures of common beverage of each type; beer, white spirits, whisky, local beverage, wine, wine coolers and vodka (can choose one type of beverage).
	5. How often did you usually have … (for each unique combination of situation(s), location(s), partner(s), and beverage type(s)) … in the last three months?”	• Every day • 5 to 6 days/week • 3 to 4 days/week • 1 to 2 days/week • 1 to 3 days/3 month (can choose one frequency category).
	6. On those days when you had …(for each unique combination of situation(s), location(s), partner(s), beverage type(s), and frequency categories)…, which containers did you usually use?	The interviewer shows pictures of various kinds of containers to the interviewee (can choose one drinking container type).
	7. And, how much did you usually have … (for each unique combination of situation(s), location(s), partner(s), beverage type(s), frequency categories, and container type(s))… per day in that container?	Answered in terms of the number of containers (can answer only one number).
These seven questions were asked in a loop for five common situations** (i.*e*., *usual drinking*, *holiday*, *party*, *cultural event*, *and music/sport event)*		

### Alcohol consumption index measures

In this study, we investigated alcohol consumption at two levels of analysis: the individual level and the sample level.

For the individual level analysis,

- **“Total consumption”** was calculated for each participant in grams of pure alcohol per three months (g/3 months). The two instruments have different methods to estimate the total consumption. Regarding BSQF, the midpoint was used to represent each frequency level. For example, “1 to 2 days/week” level was converted to 1.5 days/week or 1.5 × 13 = 19.5 days/three months. The sum of the midpoint frequencies multiplied by the quantities for all types of beverages reflected the total consumption in the last three months. The quantities can be determined by multiplying the percentage volume of pure alcohol (i.e. 5% for beer, 6% for wine coolers, 13% for wine, and 40% for white spirit, whisky, local beverage, and vodka based on the local market) and volume of beverage consumed (in milliliters), and then multiplying by 0.789 (the specific gravity of ethyl alcohol). The container size was converted into milliliters based on the standard size of alcoholic beverage containers popularly used in Thailand (i.e. 1 regular beer can = 330 mL, 1 small whisky cup = 300 mL, 1 regular cup = 50 mL). The volume consumed was calculated by the container volume (item 3) multiplied by the actual number of those containers (item 4). For CSQF, the total consumption was the sum over all situations of the product of consumption amount and frequency in the last three months for each situation.

For sample level analysis, the alcohol consumption was assessed in three consumption indices [27].

- **“Average daily intake”** was a measure of the average quantity of consumption per day (g/drinker/day) of average drinkers. It was calculated by the average “total consumption” in three months divided by 92 days.
- **“3-month per drinker consumption”** was a measure of the average amount of alcohol consumed in grams of pure alcohol by each drinker during the last 3 months (g/drinker/3 months). This was obtained from the sum of “total consumption” of all drinkers divided by the number of drinkers.
- **“3-month per capita consumption”** was a measure of the amount of alcohol consumed in grams of pure alcohol in each given sample that included non-drinkers (g/capita/3 months). It was calculated by the sum of “total consumption” of all drinkers divided by the number of all respondents.

### Validity testing

In this study, we applied the convergent validity method because the Thai Revenue Department reported total alcohol taxation only at the national level. In our study setting, taxation information is limited.

### Statistical analysis

The statistical analysis included both continuous and categorized variables for alcohol consumption indices measured by the BSQF and CSQF instruments. The median and interquartile range (IQR) were used to describe consumption indices as the alcohol consumption data were not normally distributed. The Wilcoxon signed-rank test was used to compare the alcohol consumption indices from different instruments within the same participant. Categorical variables were analyzed using a Chi-square or Fisher's exact test.

Linear regression analysis was used to identify the effects of the questionnaire variables (i.e. quantity and frequency ratio) between the CSQF and BSQF associated with calculated total alcohol consumption. Associations were expressed as standardized regression coefficients and partial regression coefficients. We used standardized regression coefficients to compare the effect size of logarithm-transformation ratios of drinking parameters between CSQF and BSQF. A logarithm-transformation was used to transform the skewed data to a symmetrical distribution more appropriate to the model. Only independent variables were standardized. They were transformed by subtracting the mean and dividing by the standard deviation. The standardized coefficient estimated the change in outcome associated with one standard deviation increase in the corresponding predictor variable.

All P values were two-tailed and significance was set at less than 0.05. All analyses were conducted using R version 3.3.2 with the epicalc [28] and the ggplot2 [29] contributed packages.

### Data visualization

**Fig 1:**
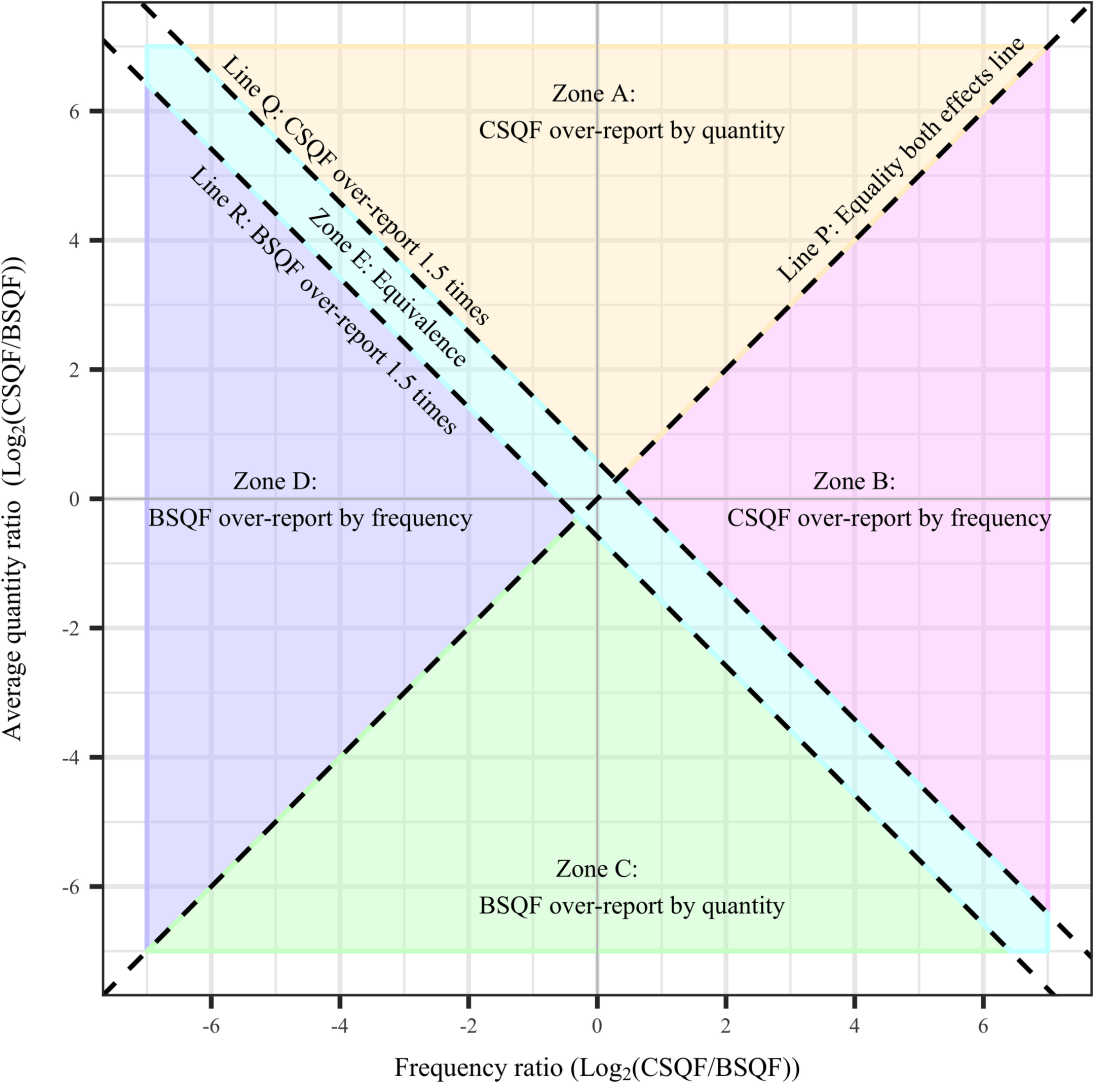
Anatomy of jitter plot of frequency ratio and average quantity ratio with five zones.

Fig 1 depicts the structure of a plot of the relationship between the logarithm of the drinking frequency ratio (X-axis) and the logarithm of the average quantity ratio (Y-axis) between the CSQF and BSQF instruments. The data are plotted in this structure as individual jittered points. The plot is jittered to clearly show individual points. Three lines divide the area into five zones.

In this paper, the terms “over-report” and “under-report” refer respectively to a higher and lower estimated level of drinking parameters by the specified instrument compared with the other instrument.

Line P is the “equality of effects line” which means that the over- or under-report between instruments is affected equally by both drinking frequency and average quantity. Line Q indicates that the total consumption reported by CSQF is more than 1.5 times higher than the BSQF while Line R indicates the opposite (i.e. the total consumption reported by BSQF is about 1.5 times higher than the CSQF).

Logarithm base 2 was applied to simplify the interpretation, in which a unit increase represents a ratio of 2. For example, if Log_2_ (CSQF _quantity_ /BSQF _quantity_) = 1, then the CSQF over-reported the average quantity compared with the BSQF 2^1^ = 2 times.

Participants in Zones A and B were those whose total consumption was over-reported by the CSQF, whereas participants in Zones C and D were those over-reported by the BSQF. Line P separates Zone A from B and Zone C from D. Zones A and C are where the discrepancy in the average quantity was greater than the discrepancy in frequency. Likewise, in Zones B and D, the discrepancy in frequency was greater than the discrepancy in the average quantity. A ratio of BSQF/CSQF or CSQF/BSQF ≤ 1.5 or within 1.5 times was considered to be within the range of equivalence and was represented in the figure by the area between Lines Q and R (Zone E).

### Ethical consideration

The study was approved by the ethics review committee for research on human subjects of the Faculty of Medicine, Prince of Songkla University (Ref no: 59-254-18-1). All of the researchers conducted the research according to the principles expressed in the Declaration of Helsinki. The objectives, benefits, and harms of the study were explained verbally and in written form to the potential participants. Written informed consent approved by the ethics committee was obtained from all study participants or parents/guardians of participants aged less than 18 years.

## Results

### Respondent characteristics

Among 804 participants, 183 (22.8%) had a history of drinking alcohol in the last three months. Most were male, aged 35 to 60 years, Buddhist, married, and had attained a primary school level of education. Most worked in agriculture and had a monthly income of between 10,000 and 26,500 Baht (30 Baht = 1 USD). About half were current smokers who started smoking at the age of 18 and had a smoking history of approximately 10 pack-years (Table 2).

**Table 2:** Characteristics of the sample by drinking status (n = 804).

Characteristics	Non-current drinker* (n = 621), n (%)	Current drinker (n = 183), n (%)	p-value
**Gender**			
Male	169 (27.2)	156 (85.2)	< 0.001_a_
Female	452 (72.8)	27 (14.8)	
**Age–**Median (IQR)	52 (40–63)	47 (35–60)	< 0.001_b_
15–29	59 (9.5)	26 (14.2)	0.035_a_
30–44	157 (25.3)	54 (29.5)	
45–59	197 (31.7)	56 (30.6)	
60–69	116 (18.7)	34 (18.6)	
70–79	66 (10.6)	12 (6.6)	
80+	26 (4.2)	1 (0.5)	
**Religion**			
Buddhism	616 (99.2)	182 (99.5)	1.00_c_
Islam	5 (0.8)	1 (0.5)	
**Marital status**			
Married	497 (80)	149 (81.4)	0.023_a_
Single	74 (11.9)	29 (15.8)	
Widowed/divorced/separated	50 (8.1)	5 (2.7)	
**Education level**			
No formal education	40 (6.4)	3 (1.6)	< 0.001_a_
Primary school	349 (56.2)	83 (45.4)	
Junior high school	75 (12.1)	27 (14.8)	
Senior high school	57 (9.2)	31 (16.9)	
Vocational certificate	43 (6.9)	17 (9.3)	
Bachelor and above	57 (9.2)	22 (12.0)	
**Occupation**			
Laborer	85 (13.7)	34 (18.6)	< 0.001_a_
Agriculture	225 (36.2)	83 (45.4)	
Commercial	65 (10.5)	11 (6.0)	
Student	28 (4.5)	17 (9.3)	
Unemployed	164 (26.4)	16 (8.7)	
Others	54 (8.7)	22 (12.0)	
**Income level (Baht/month)** –Median (IQR)	10,000 (6,000–20,000)	15,000 (10,000–26,500)	< 0.001_b_
< 5,000	93 (15.0)	17 (9.3)	0.008_a_
5,000–9,999	151 (24.3)	27 (14.8)	
10,000–19,999	178 (28.7)	64 (35.0)	
20,000–29,999	99 (15.9)	32 (17.5)	
30,000–39,999	52 (8.4)	22 (12.0)	
≥ 40,000	48 (7.7)	21 (11.5)	
**Smoking status**			
Non-smoker	510 (82.1)	73 (39.9)	< 0.001_a_
Ex-smoker	29 (4.7)	24 (13.1)	
Current smoker (≥1 day/week)	20 (3.2)	9 (4.9)	
Current smoker (<1 day/week)	62 (10.0)	77 (42.1)	
**Age at onset of smoking** –Median (IQR)	18 (15.5–20.0)	18 (16.0–20.0)	0.287_b_
**Pack-years of smoking** –Median (IQR)	11 (3.8–21.9)	10.8 (4.4–20.0)	0.903_b_

* Non-current drinkers included lifetime abstainers and former drinkers (those who drank but had not drunk during the previous three months).

_a_ Chi-square test

_b_ Wilcoxon rank-sum test

_c_ Fisher’s exact test

#### Comparisons of consumption indices between BSQF and CSQF within individuals

Comparisons of drinking frequency, average quantity, and total consumption ratio are presented in Table 3. The CSQF instrument over-reported the average quantity in 39% to 50% of current drinkers, whereas the BSQF over-reported in 7% to 23%. More than half of the participants reported equivalent drinking frequency by the BSQF and CSQF: 62.2% for beer, 75.0% for white spirits, 55.6% for whisky, and 60.7% for other beverages.

**Table 3:** Comparisons of frequency, average quantity, and total consumption ratios reported using different instruments (CSQF and BSQF) within the same participant.

Comparison instrument by beverage	Alcohol consumption parameter, n, (%)			
	(1) Average quantity ratio	(2) Frequency ratio	(3) Total consumption ratio	Number (%) of respondents over-reporting total consumption _a_
**1. Beer**				
CSQF over-report _b_	38 (42.2)	29 (32.2)	42 (46.7)	31 (73.8) vs 10 (23.8) vs 1 (2.4)
BSQF over-report _c_	21 (23.3)	5 (5.6)	21 (23.3)	17 (81.0) vs 4 (19.0) vs 0
Equivalence _d_	31 (34.4)	56 (62.2)	27 (30.0)	-
Total	90	90	90	
**2. White spirits**				
CSQF over-report _b_	12 (42.9)	5 (17.9)	14 (50.0)	11 (78.6) vs 2 (14.3) vs 1 (7.1)
BSQF over-report _c_	2 (7.1)	2 (7.1)	4 (14.3)	2 (50.0) vs 2 (50.0) vs 0
Equivalence _d_	14 (50. 0)	21 (75.0)	10 (35.7)	-
Total	28	28	28	
**3. Whisky**				
CSQF over-report _b_	41 (50.6)	26 (32.1)	41 (50.6)	26 (63.4) vs 11 (26.8) vs 4 (9.8)
BSQF over-report _c_	6 (7.4)	10 (12.3)	10 (12.3)	5 (50.0) vs 5 (50.0) vs 0
Equivalence _d_	34 (42.0)	45 (55.6)	30 (37.0)	-
Total	81	81	81	
**4. Others (local beverage, wine, wine coolers or vodka)**				
CSQF over-report _b_	11 (39.3)	3 (10.7)	10 (35.7)	10 (100.0) vs 0 vs 0
BSQF over-report _c_	2 (7.1)	8 (28.6)	8 (28.6)	1 (12.5) vs 7 (87.5)
Equivalence _d_	15 (53.6)	17 (60.7)	10 (35.7)	-
Total	28	28	28	

_a_ average quantity (Zones A, C) vs frequency (Zones B, D) vs both effects (Line P)

_b_ parameter reported by CSQF is more than 1.5 times higher than the BSQF [Log_2_ (CSQF/BSQF) > 0.58; CSQF/BSQF > 1.50]

_c_ parameter reported by BSQF is more than 1.5 times higher than the CSQF [Log_2_ (CSQF/BSQF) < -0.58; CSQF/BSQF < 1/1.50]

_d_ parameter reported by CSQF or BSQF is within 1.5 times that of the other instrument [-0.58 ≤ Log_2_ (CSQF/BSQF) ≤ 0.58; 1/1.50 ≤ CSQF/BSQF ≤ 1.50]

Regarding total consumption, over-reports by the CSQF were found in approximately 50% of current drinkers for almost all types of beverages except for “other beverages” comprising local beverage, wine, wine coolers or vodka (46.7% for beer, 50.0% for white spirits, 50.6% for whisky, and 35.7% for other beverages). The over-report of total consumption by the CSQF was mainly attributable to over-reported average quantity (average quantity effect vs. frequency effect: 73.8% vs. 23.8% for beer, 78.6% vs. 14.3% for white spirits, 63.4% vs. 26.8% for whisky, and 100% vs. 0% for other beverages). Interestingly, less than 30% of current drinkers reported higher average quantity, drinking frequency or total consumption by the BSQF instrument.

**Fig 2:**
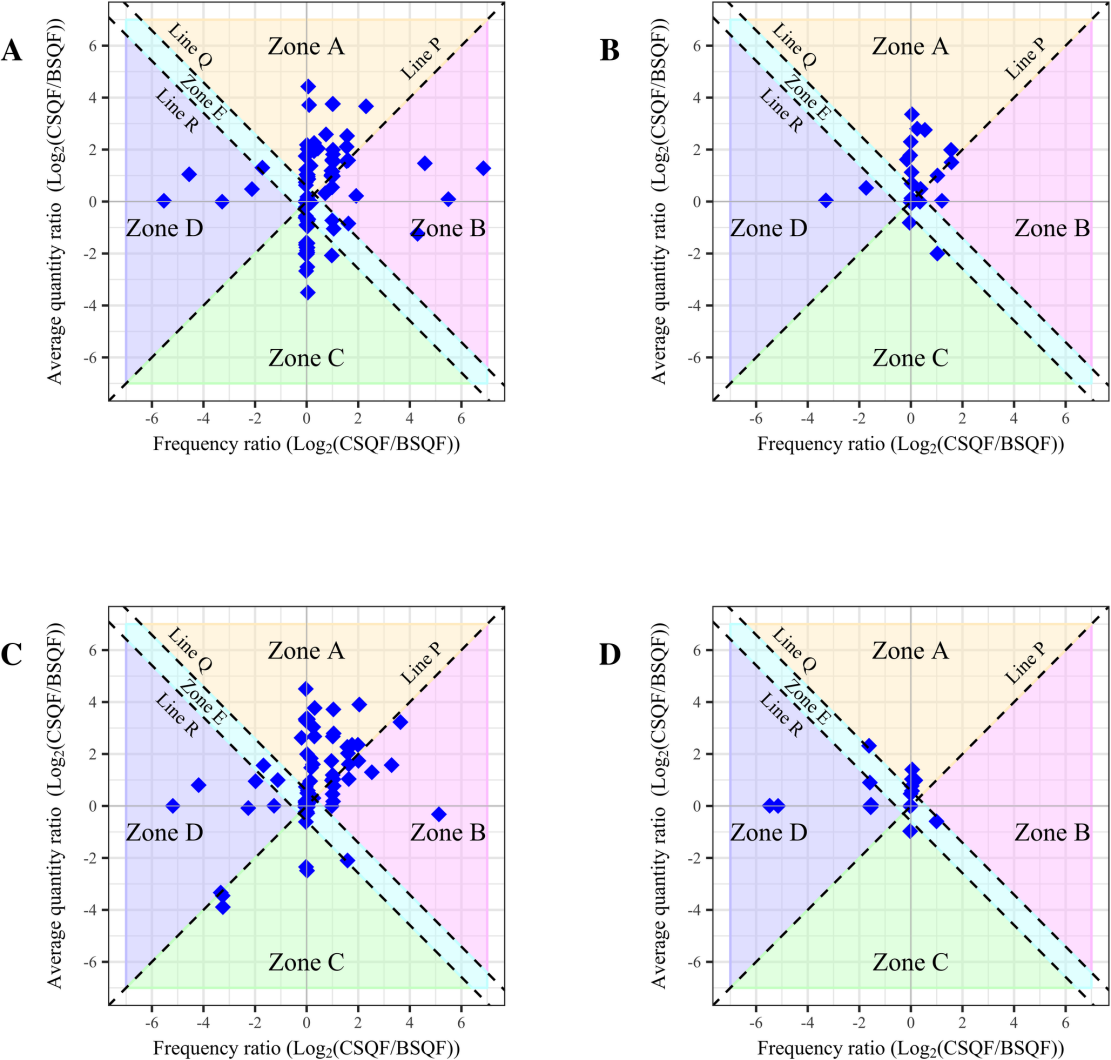
**Jitter plots of drinking frequency ratio and average quantity ratio for beer (2A), white spirits (2B), whisky (2C), and other beverages (2D)** Zone A represents CSQF over-report by average quantity, Zone B represents CSQF over-report by frequency, Zone C represents BSQF over-report by average quantity, Zone D represents BSQF over-report by frequency, and Zone E represents equivalence; Line P represents the equality of effect line, Line Q represents CSQF over-report of 1.5 times, and Line R represents BSQF over-report of 1.5 times.

Fig 2 has four jitter plots for specific beverages depicting the relationships between drinking frequency ratio (X-axis), quantity ratio (Y-axis), and total consumption (CSQF over-report in Zones A and B and BSQF over-reports in Zones C and D, with equivalence in Zone E). These figures visualize the complex results from Table 3. Fig 2A, 2B and 2C highlight the preponderance of points in the CSQF over-report areas (Zones A and B) with more in Zone A (average quantity over-report) than in Zone B (frequency over-report).

#### Effects of CSQF-BSQF quantity and frequency ratios on total consumption ratios

The ratios of CSQF to BSQF total consumption could be explained more by the discrepancies in drinking frequency reported by the two methods for most types of beverages than by the discrepancies in drinking quantity (beta of frequency ratio vs. average quantity ratio = 1.309 vs. 1.099 or 2^1.309^/2^1.099^ = 1.16 times for beer, 1.02 times for whisky and 1.84 times for other beverages) except for drinking of white spirits (beta of frequency vs. average quantity ratio = 0.759 vs. 0.978 or 2^0.759^/2^0.978^ = 0.86 times). In a precise sense it revealed that a one standard deviation increase in the frequency variable ratio (as Log_2_) between the instruments implied an expected difference of 2^1.309^ = 2.48 times of ratio difference in the total consumption of beer, whereas a one standard deviation increase in the average quantity ratio (as Log_2_) implied only 2^1.099^ = 2.14 times of ratio difference in the total consumption of beer. The partial correlation coefficient trends in Table 4 are in conformity with the standardized regression coefficients.

**Table 4:** Multiple linear regression analysis of the Log_2_ ratio of total consumption (dependent variable) against Log_2_ ratio of frequency and Log_2_ ratio of average quantity (independent variables) between the CSQF and BSQF.

Variable	Standardized coefficient		Unstandardized coefficient				Partial correlation
	Beta	p-value	B	se	t value	p-value	
**1. Beer**							
Intercept	0.197	0.009	-0.414	0.077	-5.366	<0.001	
Frequency ratio between CSQF and BSQF (Log_2_)	1.309	<0.001	0.850	0.048	17.598	<0.001	0.884
Average quantity ratio between CSQF and BSQF (Log_2_)	1.099	<0.001	0.716	0.048	14.781	<0.001	0.846
	R^2^ = 0.877, Adjusted R^2^ = 0.874						
**2. White spirits**							
Intercept	0.295	0.033	-0.373	0.153	-2.443	0.022	
Frequency ratio between CSQF and BSQF (Log_2_)	0.759	<0.001	0.830	0.147	5.663	<0.001	0.750
Average quantity ratio between CSQF and BSQF (Log_2_)	0.978	<0.001	0.821	0.112	7.299	<0.001	0.825
	R^2^ = 0.796, Adjusted R^2^ = 0.780						
**3. Whisky**							
Intercept	0.427	<0.001	-0.446	0.078	-5.724	<0.001	
Frequency ratio between CSQF and BSQF (Log_2_)	1.406	<0.001	0.923	0.050	18.415	<0.001	0.902
Average quantity ratio between CSQF and BSQF (Log_2_)	1.375	<0.001	0.832	0.046	18.014	<0.001	0.898
	R^2^ = 0.933, Adjusted R^2^ = 0.932						
**4. Others (local beverage, wine, wine coolers or vodka)**							
Intercept	-0.896	<0.001	-0.417	0.136	-3.069	0.007	
Frequency ratio between CSQF and BSQF (Log_2_)	1.464	<0.001	0.859	0.062	13.772	<0.001	0.958
Average quantity ratio between CSQF and BSQF (Log_2_)	0.583	<0.001	0.786	0.143	5.486	<0.001	0.799
	R^2^ = 0.933, Adjusted R^2^ = 0.926						

#### Comparisons of alcohol consumption indices of the whole sample between CSQF and BSQF

A summary of the alcohol consumption indices for each instrument is presented in Table 5. There was no significant difference in the average daily intake, 3-month per drinker consumption or 3-month per capita consumption between instruments in the sample level analysis. However, the CSQF provided drinking contexts which the BSQF did not, while the interview duration and the burden of the participants to answer the questions for the CSQF were not significantly higher than those for the BSQF. The median time actually spent answering the instrument was 3 (interquartile range [IQR], <1 to 3) minutes for CSQF and 2 (IQR, <1 to 2) minutes for BSQF. The burden of the participants placed on both instruments was rated at 2 (IQR, 1 to 2) from a total score of five.

**Table 5:** Summary drinking variables by measurement instruments (BSQF and CSQF; n = 804 with 183 current drinkers).

Alcohol indices and others	CSQF	BSQF	Median difference (95% CI) _a_
**Drinking indices**			
**Average daily intake (n = 183)** (g/drinker/day), Median (IQR)	8.66 (3.11–27.34)	7.54 (2.36–24.61)	0.56 (-0.30, 2.50)
** 3-month per drinker consumption (n = 183)**	796.32 (286.18–2,515.46)	693.23 (217.15–2,264.53)	51.82 (-27.93, 229.89)
(g/drinker/3 months), Median (IQR)			
** 3-month per capita consumption (n = 804)**	472.85 (1,651.41)	412.77 (1,550.92)	51.82 (-27.93, 229.89)
(g/capita/3 months), Mean, (SD)			
**Interview duration (n = 183)** (minute), Median (IQR)	*3 (<1–3)*	*2 (<1–2)*	1.00 (0, 1.00)
**Participation's burden (n = 183)** (total score = 5), Median (IQR)	*2 (1–2)*	*2 (1–2)*	1.00 (0, 1.00)

_a_ Wilcoxon signed-rank test

## Discussion

### Summary of results

The present study aims to compare the CSQF and BSQF for estimating alcohol consumption indices at the individual (i.e., total alcohol consumption) and sample levels (average daily intake, 3-month per drinker, and 3-month per capita). An instrument used in an alcohol survey should provide as accurately as possible the consumption indices. Several methodological issues influence the accuracy such as reference period, beverage-specific versus overall approach, open-ended versus categorical pattern, standard versus actual drink sizes, and interviewing methods (face-to-face versus telephone/computerized instruments) [30]. This study focused on the contextual approach technique.

To the best of our knowledge, this is the first study to compare the contextual approach method (i.e. the CSQF instrument) and the BSQF instrument to assess alcohol consumption at individual and sample levels. We found that asking about the volume of alcohol consumption specific to the context, including situation, place, and partner, provided higher consumption volume in the past three months compared to the standard BSQF method, while the interview duration and burden on the participant to answer the questions were not significantly higher. This is in keeping with previous findings that motivation and a location-specific approach can estimate higher total consumption in the previous week than the traditional QF or recent occasion methods [20]. Questions asking about the most typical locations or occasions of drinking also provided a higher total alcohol consumption than did the QF, L7D, and two recent occasions methods [17].

We also found that the quantity of consumption contributed to the difference of total consumption measured by the two instruments. The higher volume of consumption reported by the CSQF might be because the context-specific questions increased the recall ability by stimulating the respondents to think of all the different situations they consumed alcohol [31], whereas the BSQF could only capture usual or average drinking events.

However, in terms of variability, our study revealed that variation in drinking frequency had a greater effect on the ratios of CSQF to BSQF total consumption than the average quantity. The variability of frequency categories and time frame is one important dimension for alcohol consumption assessment [32]. In this study, we measured the average quantity of drinking using open-ended questions based on the number of containers the drinkers usually took for drinking (e.g., glass, cup, bottle, and can). On the other hand, drinking frequency was based on a ordinal item as it was reported to provide easier, higher alcohol consumption estimates and less item-missing data than reporting in an open-ended question [33]. However, it might be that the frequency categories we used, which were based on those used in other instruments, might not capture all drinking frequencies by all groups of drinkers. Thus, we suggest subdividing the frequency category into more categories. For example, adding “2 to 3 days/month (every fortnight)” to fill the gap between “1 to 2 days/week (every week)” and “1 to 3 days/3months (every month)”.

### Strengths and limitations

This study has several strengths. First, using the same retrospective time frame in both instruments possibly minimized the measurement errors from adjusted drinking frequencies. A past-year reference period was previously suggested to link a alcohol drinking pattern with alcohol-related harm [34]. A 12-month time frame is recommended by some studies because it is appropriate for drinking cultures where alcohol is used seasonally or influenced by various festive activities [27, 34]. The 12-month time frame attributes to usual drinking more than a detailed memory of actual drinking events [30]. Hence, a 3-month time frame was applied in this study because we supposed that it would be the average timeframe over which most drinkers would be able to remember their drinking history with less recall bias effect. This 3-month reference period covered (i) usual days, (ii) Christmas and New Year’s Day (celebration), (iii) Constitution Day (holiday), and (iv) Buddhist Lent and a Thai festival at the end of 10^th^ lunar month (cultural event). These days commonly have different drinking situations in Thailand. In Thailand, New Year’s Day and the Buddhist Lent are the periods of greatest and lowest alcohol consumption, respectively. Second, both individual and sample level analyses were done in this study. An accurate estimate at the individual level would facilitate an accurate estimate at the sample level. Last, the actual time and burden in responding to the questionnaires were measured. An increased response burden may result in a low response rate, incomplete questionnaire, and reduced data quality. One important questionable disadvantage of the contextual approach is a greater response burden because of longer and more complex questions [21]. Based on the guideline for Minimizing Perceived Respondent Burden, response burden can be divided into actual and perceived burdens [35]. In this study, the actual and perceived response burden in completing the CSQF was not significantly higher than that of the BSQF in either dimension. This finding was consistent with a meta-analysis study revealing weak support for an association between questionnaire length and response burden in medical and public health questionnaires [36].

We also acknowledge that our study may have some limitations. It is generally known that there is no definite “gold standard method” to estimate alcohol consumption and validate a new instrument such as the CSQF [23, 24]. Researchers typically want the criterion validity to be measured against a gold standard, but the convergent validity method is another powerful method which was applied in this study because there is no specific gold standard to assess alcohol consumption. Prospective data collection can be more accurate in measuring alcohol drinking history using a self-recorded diary, mobile application or telephone interview by trained staff [37]. Since prospective data collection was not a feasible method in our study sample, a retrospective inquiry of consumption in the previous 3-month period was used instead to minimize recall bias, and the comparison between two instruments was reported rather than a comparison with a “gold standard”. Second, drinking situations in the CSQF are not mutually exclusive. Although the CSQF provides examples of each drinking situation to minimize the double counting effect, some participants were confused concerning the situation categories (e.g., drinking at a New Year’ party can be considered as drinking on a holiday or during a celebration). Therefore, the CSQF-over-report could be explained by this double counting. Third, both the CSQF and BSQF assess the same construct, which is the amount of alcohol consumption and they both have some identical questions. This may overestimate the concordance between the two measures and limit the chance that occasional influencing factors affect self-reports. However, both the CSQF and BSQF in our study asked about the consumption in the same time frame of the past three months, which is a relatively short period. It was not possible to separate the interview into two occasions at 2–3 days apart. Fourth, the actual number of drinking days could not be accurately estimated by either instrument. The actual number of drinking days is an important variable to calculate “drinking intensity” which has many clinical benefits. However, the main purpose of the CSQF development is for public health implication. Fifth, when population-level indices were compared, there were no significant differences between the two instruments. This might be due to the small number of drinkers in this study, which resulted in insufficient power to reveal the significant differences by the Wilcoxon Signed-Rank Test. Last, the generalizability of this study is limited by the small–scale, localized single population which possibly has culture-specific drinking patterns. The alcohol consumption level and drinking patterns have high variability among WHO regions due to different drinking cultures and contexts [38]. The WHO Eastern Mediterranean Region (EMR) and South-East Asia Region (SEAR) including Thailand are regions of the lowest consumption levels and most drinkers are occasional drinkers (less than one day per week). In contrast, in other regions there are high levels of alcohol consumption and most drinkers are regular drinkers [39]. However, our aim was to initially test the hypothesis on a small scale. Had we found a significant result, we would draw a sample from many provinces in a further study. Nevertheless, this localized study has provided information with important implications for alcohol-related policy at the particular site.

### Implications and further studies

The findings of our study have considerable managerial implications for the health-care sector and the alcohol survey manager who will select the appropriate instrument to estimate alcohol consumption in each survey. A full picture of drinking behaviors from the CSQF has several valuable advantages. Specific alcohol policies can be more directly specific to some target populations or situations. For example, if strategies to prevent underage drinking are launched, the CSQF can provide the specific conditions such as the occasion (when), location (where), partner (with whom), and types of beverages (what), that are strongly associated with underage drinking. Consequently, alcohol specified-group rules or interventions can be framed.

Our suggestions for CSQF users are to use a technology-assisted technique such as personal cellphones, functionalities (e.g., text, calls, internet, GPS, sound recorders, and applications), skipping function or sequence of items to minimize human errors caused by the complexity of the questionnaire and to ask questions in a loop within each context to ease recall. Technology and other innovative ways for data collection purposes in alcohol research have many advantages (e.g., matching date, location via GPS with alcohol consumption, possibility of response to previous answers, enhancing repeated measurements, and minimizing recall bias [40–42]. Categorical responses should be modified to suit each country in terms of drinking cultures such as local beverage types, cultural or regional events or containers.

Lastly, additional methodological studies are needed to further explore the inter-interviewer reliability and test-retest reliability of the instruments using the same retrospective timeframe. The acceptability in multiple languages and cultures needs to be demonstrated in the future. Data collection from taxable alcohol available for consumption is another useful source to validate the survey results and can be used for cross-country comparison [22, 27]. However, in our study we could not obtain the taxation data.

### Conclusions

The inclusion of drinking context in harm reduction surveys is recommended. The CSQF appears to be appropriate for an alcohol consumption survey because it provides significantly higher total alcohol consumption than the BSQF at the individual level and provides drinking contexts (situation, place, and partner), which are not part of the BSQF. The major effect of the difference between two instruments was the over-reporting of average quantity. However, there was no significant difference in the average daily intake, 3-month per drinker consumption or 3-month per capita consumption between instruments in the sample level analysis. The interview duration and participant’s perceived burden to answer the questions for the CSQF were not significantly higher than those for the BSQF.

The methodological research on measuring alcohol consumption generally values the instrument which estimates the highest alcohol consumption. However, an instrument which captures drinking context can provide more useful information with public health implication than the one that simply estimates the highest alcohol consumption indices.
